# Recombinant immunotoxin anti-c-Met/PE38KDEL inhibits proliferation and promotes apoptosis of gastric cancer cells

**DOI:** 10.1186/1756-9966-30-67

**Published:** 2011-07-07

**Authors:** Xu Wei, Zhu Xiao Juan, Feng Xiao Min, Cai Nan, Zhang Xiu Hua, Feng Zheng Qing, Liu Zheng

**Affiliations:** 1 Department of Gastroenterology, The Second Affiliated Hospital of Nanjing Medical University, Nanjing, 210029, PR China; 2 Department of Pathology, Nanjing Medical University, Nanjing, 210029, PR China

## Abstract

**Background:**

Our study aims to evaluate the anti-growth effects of recombinant immunotoxin (IT) anti-c-Met/PE38KDEL on gastric cancer cells, and its mechnisms.

**Methods:**

Gastric cancer cells were treated with increasing doses of IT and c-Met protein was quantified by Western blotting. Cell proliferation was determined by Cell Counting Kit-8 assay (CCK). [^3^H]-leucine incorporation assay was used to evaluate IT inhibition of protein synthesis. Cell apoptosis was quantified by flow cytometry. Caspase activities were measured using colorimetric protease assays.

**Results:**

Cell growth and protein synthesis of the gastric cancer cell lines were suppressed by IT in a dose- and time-dependent manner. IT also induced apoptosis in a dose-dependent manner. The apoptosis rates of gastric cancer cell lines MKN-45 and SGC7901 were 19.19% and 27.37%, respectively when treated with 50 ng/ml of IT. There were significant increase ofcaspase-3 activity at 24 hr of IT treatment (100 ng/ml) (P < 0.01) in these gastric cancer cell lines.

**Conclusions:**

IT anti-c-Met/PE38KDEL has anti-growth effects on the gastric cancer cell lines *in vitro*, and it provides an experimental basis for c-Met-targeted therapy towards *in vivo *testing.

## Introduction

Gastric carcinoma (GC) is one of the most common and lethal malignant cancers [1]. Despite the improving surgical techniques and new chemotherapeutic treatment regimens, the patient survival rate remains dismal [2], and effective alternative treatment approach is in vital need. GC has been shown to harbor multiple somatic mutations as well as over-expressions of oncoproteins. Identification of these GC-associated biomarkers may entail possible discovery of new therapeutic targets [3]. Among various GC-associated biomarkers, c-MET gene is frequently found gnomically-amplified and over-expressed in GC cell lines [4]. The proto-oncogene c-MET, a receptor of hepatocyte growth factor (HGF, also known as scatter factor), encodes a 190 kDa heterodimeric transmembrane tyrosine kinase. HGF binding to c-Met triggers tyrosine kinase domain auto-phosphorylation and induces pleiotropic responses such as proliferation, motility, morphogenesis and angiogenesis in many cell types including normal and tumor cells [5]. c-MET amplification has been identified in nearly 74% of human GC specimens [6]. HGF and c-MET both play important roles in the progression and metastasis of GC [7]. Thus, c-Met has been considered as a promising therapeutic target for various cancers.

Immunotoxins (ITs) are fusion proteins composed of a toxin fused to an antibody or growth factor with distinct target specificity [8]. IT exerts its anti-growth effect by inhibiting protein synthesis and promoting apoptosis [9]. IT anti-c-Met/PE38KDEL (anti-c-Met Fab, which resulted from screening and characterization from a natural human Fab phage antibody library; PE38KDEL, which is a modified structure of PE38, lost the function of combining with non-mammalian cells specifically, but retained a complete cytotoxicity after internalization) has shown specific cytotoxic effects against c-Met-positive cancer cells [10]. In this study, we investigated the effects of IT anti-c-Met/PE38KDEL on proliferation and apoptosis of two different c-Met-positive malignant gastric cell lines, MKN-45 and SGC7901 [11,12], and a normal gastric mucosa cell GES-1 [13]. We found that IT anti-c-Met/PE38KDEL exerts its anti-growth effect primarily through rapid inhibition of protein synthesis.

## Materials and Methods

### Immunotoxin

IT anti-c-Met/PE38KDEL was described previously [9]. It induces apoptosis in hepatic carcinoma cells SMMC7721. Cell Counting Kit 8 (CCK8) was purchased from Sigma Chemical. Caspase colorimetric assay kit and anti-caspase-3 antibody were from Biovision. Antibodies against c-Met and β-actin were purchased from Santa Cruz. Protein lysis buffer was from TaKaRa Biotechnology.

### Cell culture

GC cells lines, MKN-45 and SGC7901, and normal gastric mucosa cells GES-1 were obtained from the Cell Bank of Type Culture Collection of the Chinese Academy of Sciences (Shanghai, China), and were grown in DMEM (Invitrogen) supplemented with 10% fetal calf serum (FCS) and incubated at 37°C with 5% CO_2_. All cell lines were routinely tested and found to be free from mycoplasma contamination.

### Western Blotting

GES-1, MKN-45 and SGC7901 cells grown in 6-well plates were collected in lysis buffer for total cellular protein. Protein concentrations were measured using a Bradford reagent (Bio-Rad). Equal amounts of protein (80 μg/lane) from each cell line were boiled for 5 min, separated by SDS-PAGE, and then transferred on to a nitrocellulose membrane before blocking in 5% non-fat dried milk in Tris-buffered saline (TBS) for 120 min at room temperature. The membranes were then incubated with a primary anti-human c-Met polyclonal antibody (diluted 1:150 in a new batch of the blocking buffer) or a goat polyclonal primary anti-β-actin (diluted 1:1000, Santa Cruz, CA, USA) for 2 hr and followed by incubation with peroxidase-labelled anti-IgG secondary antibody for 1 hr. After washing with TBST for 3 times, the films were developed and the protein bands were quantified by densitometry using ImageJ software (NIH, Bethesda, MD, USA).

To detect the caspase-3 activity, both floating and adherent cells were collected 24 hr following IT treatment. Total cellular protein was prepared as described above. All the experiments were performed at least twice with similar results.

### Cell proliferation assay

Cell growth inhibition rate (IR) was determined using a CCK- 8 assay following the manufacturer instructions (Sigma). GES-1, MKN-45 and SGC7901 cells were seeded at a concentration of 1 × 10^5 ^cells/90 μl/well in 96-well culture plates. After incubation of cells with the indicated concentrations of IT for 24 hr and 48 hr, 10 μl/well of cell Counting Kit-8 solution was added to the medium and the cells were incubated for an additional 4 hr. The absorbance at 450 nm was then measured in a Microplate Reader. IR was calculated using the following equation: IR = [1-(*A *value in the treated samples-*A *value in the blank samples) / (*A *value in the control samples-*A *value in the blank samples)] *100%. The assays were performed in triplicates and repeated at least twice [14].

### Protein synthesis inhibition assay

IT-induced inhibition of protein synthesis in GES-1, MKN-45 and SGC7901 cells were evaluated using the [^3^H]-leucine incorporation assay [15]. Cells were seeded in 48-well plates (1 × 10^4 ^per well) and allowed to grow overnight before the addition of IT at different concentrations. After 5 or 24 hr incubation, cells were washed twice with cold phosphate-buffered saline (PBS) containing 0.1% FCS, and then incubated with [^3^H]-leucine (2 μCi ml^-1^) in leucine-free medium at 37°C for 45 min. Cells were then washed with 5% trichloroacetic acid (TCA) for 5 and 10 min, respectively, and dissolved in 0.1M KOH for 10-15 min. The resultant solution was transferred to the liquid scintillator. Sample counts were determined in a liquid scintillation counter. Assays were performed in duplicates and repeated at least three times. Counts per minute (cpm) for treated cells were compared to cpm for untreated cells and reported as a percentage of leucine incorporation with the control value set to 100%[16]. The experiment was completed in the isotope laboratory of Nanjing Medical University.

### Flow cytometric analysis of cell apoptosis

Apoptosis were determined by flow cytometric analysis. Briefly, cells in triplicates, were incubated with or without various concentrations of IT for 24 hr. Cells were then harvested, washed in cold PBS, and fixed with 1 ml 75% ice-cold ethanol at -20°C until processing. An aliquot (1 ml) of fixed cell suspension containing 1 × 10^6 ^cells was washed twice in cold PBS and then treated with fluorochrome DNA staining solution (1 ml) containing 40 μg of propidium iodide and 0.1 mg of RNase A in the dark at room temperature for 0.5 hr. Flow cytometric analysis were performed three times [17].

### Caspase activity assay

Caspase activity was determined in 96-well plates using cell lysates from 1 × 10^6 ^cells for each measurement. Caspase-3 and caspase-8 activities were determined using colorimetric assay kits according to the manufacturer's protocol (BioVision). GES-1, MKN-45 and SGC7901 cells were treated with anti-c-Met/PE38KDEL (100 ng/ml) for 24 hr prior to the assay. Cell extracts were incubated with 5 μl of 4 mM tetrapeptide substrates (DEVD, caspase-3; IETD, and caspase-8) at 37°C for 1-2 hr. The reaction was measured at 405 nm in a Microplate Reader. Background readings from cell lysates and buffers were subtracted from the readings of both IT-induced and control samples before calculating the relative change increase in caspase activity in the IT-induced samples compared to that of the control. IT treated samples were normalized to the caspase activity of the untreated sample, which was set to 1.0. Fold of increases in caspase activities were presented.

### Statistical analysis

Statistical analysis was performed with SPSS 13.0 software. Data were presented as mean ± standard deviation. Student's t-test was used to compare two samples, and the single-factor analysis of variance (One-way ANOVA) was used to compare multiple samples. A p-value less than 0.05 is considered statistically significant (*, p < 0.05; **, p < 0.01).

## Results

### Increased c-Met expression in MKN-45 and SGC7901 cells

**Figure 1:**
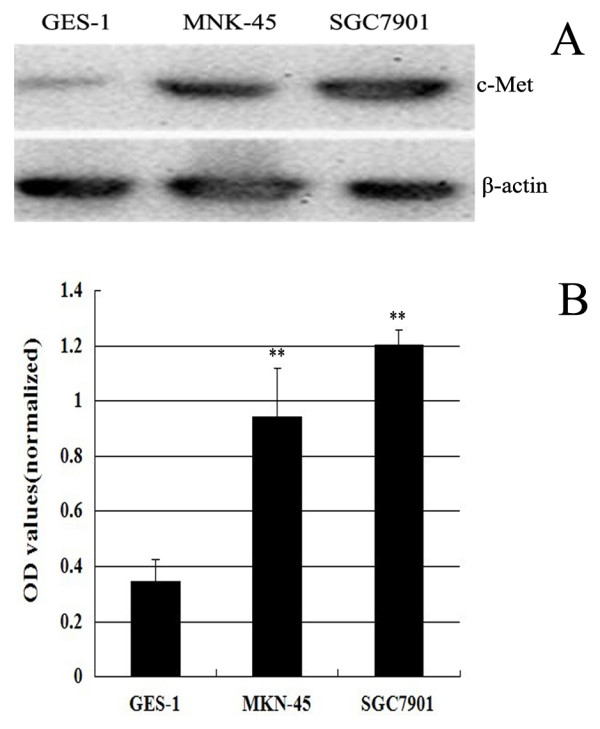
**Overexpression of c-Met in castric carcinoma cell lines**. Lysates (80 μg/lane) from normal gastric mucosa cells GES-1 and GC cell lines MKN-45 and SGC7901 were analyzed for c-Met protein level by western blot using an anti-c-Met antibody and an anti- β-actin antibody (loading control) (Figure 1A). The optical densities (OD's) of the Western blot bands were measured using Image J (Figure 1B).

To determine the c-Met protein expression levels in GC, we used western blotting to examine c-Met protein in two GC cells (MKN-45 and SGC7901) and one normal gastric mucosa cells GES-1 (Figure 1A). c-Met proteins is 3-4 fold higher in MKN-45 and SGC7901cells than GES-1 cells. SGC7901 cells express slightly more c-Met than MKN-45 cells (Figure 1B). The optical densities (OD's) of the Western blot bands were measured using ImageJ. The OD for each band was normalized to β-actin. MKN-45 and SGC7901 had a 0.94 and 1.27 fold increase in the expression of c-Met over the control, but only 0.34 fold increased in GES-1.

### IT anti-c-Met/PE38KDEL inhibited cell proliferation and protein synthesis

**Figure 2:**
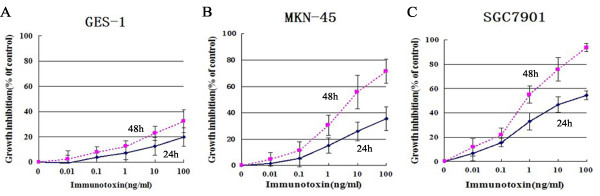
**IT anti-c-Met/PE38KDEL induced inhibition of cell proliferation**. Cell growth inhibition as a function of varying concentrations of IT (expressed as a percentage of untreated cells), Normal cell GES-1 (A), GC cells MKN-45 (B) and SGC7901 (C) were treated with various concentrations of IT for 24 hr and 48 hr.

GC cells have significantly higher c-Met protein levels than normal gastric mucosa cells, therefore we tried to determine if IT anti-c-Met/PE38KDEL has GC-specific effects. The anti-proliferative effect of IT anti-c-Met/PE38KDEL on GES-1, MKN-45 and SGC7901 cells was measured using CCK8 kit. Cells were harvested at 24 or 48 hr after IT treatment. As shown in Figure 2, IT inhibited GC cell growth in a time- and dose- dependent manner. 1, 10 and 100 ng/ml of IT caused a dramatic growth inhibition in MKN-45 and SGC7901 cells (*P*< 0.01). 48 hr of IT treatment (100 ng/ml) resulted in a growth inhibition of 30% in GES-1 cells (Figure 2A). However, inhibitions of 75% and 95% were observed in MKN-45 and SGC7901 cells (Figure 2B and 2C), respectively. Further, we found that there is a strong correlation between c-Met expression and *in vitro *immunotoxin efficacy.

**Figure 3:**
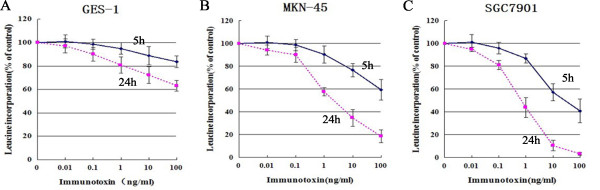
**Anti-c-Met/PE38KDEL induced inhibition of protein synthesis**. The ability of IT to inhibit protein synthesis in GES-1, MKN-45 and SGC7901 cells were evaluated by using the [^3^H]-leucine incorporation assay. [^3^H]-leucine incorporation for protein synthesis as a function of varying concentration of IT (expressed as a percentage of untreated cells), Normal cell GES-1 (A), GC cells MKN-45 (B) and SGC7901 (C) were treated with varying concentration of IT for 24 hr and 48 hr.

Given the high c-MET levels in MKN-45 and SGC7910 cell lines, we hypothesize that anti-c-Met/PE38KDEL can attenuate cancer cell growth through inhibition of protein synthesis via c-Met inhibition. The effects of anti-c-Met/PE38KDEL on protein synthesis in GES-1, MKN-45 and SGC7901 cells are shown in Figure 3. The IT's IC_50 _value on GES-1 cells was approximately 120 ng/ml. However, IT induced more potent inhibitions of protein synthesis in MKN-45 and SGC7901 cells, with IC_50 _values of 5.34 ng/ml and 0.83 ng/ml, respectively. Nearly 80% and 100% of inhibitions were observed with 100 ng/ml of IT treatment in these two GC cells (Figure 3B and 3C). In contrast, 100 ng/ml of IT only caused a 35% decrease in protein synthesis in GES-1 cells (Figure 3A). These results suggested that anti-c-Met/PE38KDEL can attenuate cell growth through the inhibition of protein synthesis.

### IT anti-c-Met/PE38KDEL inhibits tumor cell growth through induction of apoptosis

**Figure 4:**
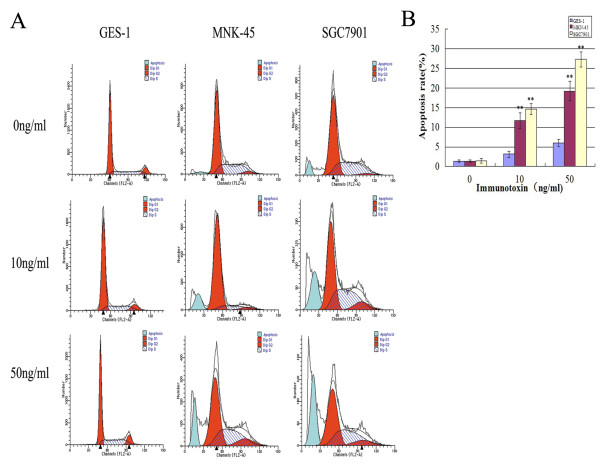
**IT anti-c-Met/PE38KDEL inhibited tumor cell growth through induction of apoptosis**. To measure the dose response effect of IT on cell apoptosis rate of GES-1, MKN-45 and SGC7901, cells were treated with different concentrations of anti-c-Met/PE38KDEL. Cells were incubated with IT at 0, 10 and 50 ng/ml for 24 hr, and the percentage of cell apoptosis was determined by flow cytometry. IT induced apoptosis for its anticancer effect.

To determine whether the anti-proliferative effect of IT was due to cell apoptosis, we used flow cytometric (FCM)) to further determine if IT induces cell apoptosis. As shown in Figure 4A and 4B, apoptotic rates in MKN-45 and SGC7901 cells were increased from 1.89% and 2.4% (0 ng/ml), to 19.19% (P < 0.01) and 27.37% (P < 0.01) (50 ng/ml), respectively. The apoptosis rate of GES-1 cells is significantly lower than two GC cells (5.98%, P < 0.01) at the IT dose of 50 ng/ml. These data indicate that anti-c-Met/PE38KDEL induced apoptosis in GC cells.

### IT anti-c-Met/PE38KDEL activates caspase-3

**Figure 5:**
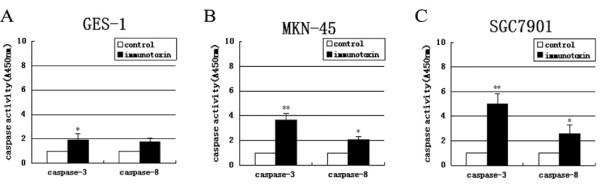
**IT anti-c-Met/PE38KDEL mainly activates caspase-3**. Caspase-3 and caspase-8 activities in GES-1 (A), MKN-45 (B) and SGC7901 (C) cells were measured in control or IT-treated cells (immunotoxin) (24 hr) using the Caspase colorimetric assay kit. * P < 0.05, **P < 0.01.

To determine whether apoptotic pathway is activated by IT in GC cells, we measured caspase-3 and caspase-8 activities following IT treatment. As shown in Figure 5B and 5C, MKN-45 and SGC7901 cells showed 3.70 and 5.02 fold of increases in caspase-3 enzyme activity as compared to untreated controls after 24 hr IT treatment (P < 0.01). GES-1 exhibited a 2.03-fold increase in caspase-3 enzyme activity (P < 0.05) (Figure 5A). Caspase-8 enzyme activity in two GC cell lines also increased (P < 0.05), suggesting caspase-3 activation mediates IT anti-c-Met/PE38KDEL-induced biological effects.

**Figure 6:**
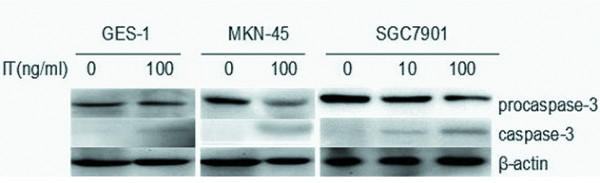
**IT-induced caspase 3 cleavage**. Lysates from normal gastric mucosa cells GES-1 and GC cell lines MKN-45 and SGC7901 with or without IT treatment were analyzed for procasoase-3 protein levels and activated caspase protein levels by western blot using an anti- procaspase-3, anti-activated caspase-3 and anti- β-actin antibodies (loading control).

The caspases are synthesized as inactive precursors (zymogens) that are proteolytically processed to generate active subunits by cleaving specific aspartic acid residues [18], and are essential for the execution process of apoptosis as effector proteases [19]. In the process of IT-inducd apoptosis, caspase-3 appeared to play a role. We investigated whether caspase-3 is regulated in anti-c-Met/PE38KDEL-induced cell death. As shown in Figure 6, procaspase-3 was proteolytically cleaved in a dose-dependent manner after 24 hr of IT treatment, resulting in the production of the active caspase-3 fragment (17 kDa). In untreated control cells (0 ng/ml), no caspase-3 was detected. All these results suggested that IT anti-c-Met/PE38KDEL causes apoptosis at least partially via activation of caspase-3.

## Discussion

GC is the second leading cause of cancer mortality in the world [20]. The receptor tyrosine kinase c-Met is constitutively activated in many GCs [2]. Amplifications of c-Met have been associated with human GC progression [21] C-Met is also related to lymph node metastasis in GC [22]. Therefore, c-Met is considered a promsing therapeutic target for this type of cancer [3]. The aim of this study was to evaluate the effects of recombinant immunotoxin anti-c-Met/PE38KDEL on proliferation and apoptosis of GC cells and explore the mechanism underlying the action of anti-c-Met/PE38KDEL.

SGC7901 was derived from moderately differentiated GC, with a high metastatic potential [23]. MKN-45 was derived from poorly differentiated GC with low metastatic potential [24]. We found that SGC7901 cells expressed high level of c-Met than MKN-45 cells. Normal gastric mucosa cells GES-1 expressed a minimum level of c-Met. Studies have shown that c-Met overexpression in carcinoma cells is associated with liver metastasis of GC [25]. Moreover; c-Met expression can be used as an indicator of liver metastasis for GC patients. It has also been reported that HGF is a lymphangiogenic factor, which can directly or indirectly stimulate lymphangiogenesis and contribute to lymphatic metastasis in GC [26]. Therefore, we hypothesized that IT anti-c-Met/PE38KDEL may be effective in preventing GC's metastasis.

Our data showed that IT decreased GC cell proliferation in a time- and dose-dependent manner. After 48 hr of IT treatment (100 ng/ml), cell inhibition rate in MKN-45 and SGC7901 cells was about 75% and 95%, but only 30% in GES-1 cells, presumably due to low c-Met expression on GES-1 than the two GC cells. IT attenuates cancer cell growth not only by inhibiting protein synthesis but also by inducing apoptosis [27]. We found that IT anti-c-Met/PE38KDEL induced a rapid inhibition of protein synthesis with simultaneous induction of apoptosis in GC cells. Nearly 80% and 100% inhibitions of protein synthesis were observed after 24 hr treatment with IT (100 ng/ml) in the MKN-45 and SGC7901 cells, respectively. The inhibition was much less pronounced in GES-1 cells (35%), suggesting that IT anti-c-Met/PE38KDEL is selective against GC. In addition, IT exerts its anticancer effect mostly via induction of cells apoptosis. The apoptosis rates in three cells were all increased after treatment with IT, more prominent in the two GC cell lines.

Caspases are classified into two functional subgroups-initiator caspases and effector caspases. The initiator caspases are caspase 2, 8, 9 and 10, and the effector caspases are caspase 3, 6 and 7 [28]. Caspases are critical mediators of apoptosis [29]. Activation of caspase is responsible for multiple molecular and structural changes in apoptosis [30]. Caspase-3 is a potent effector of apoptosis in a variety of cells [31] and plays a central role in both death-receptor and mitochondria-mediated apoptosis. Caspase-8 is the prototypical apoptosis initiator downstream of TNF super-family death receptors. Our data showed that caspase-3 enzyme activity exhibited 3.70, and 5.02 fold increases in IT-treated MKN-45 and SGC7901 cells as compared to the activity of untreated controls (P < 0.01). The increase in caspase-8 enzyme activity was less significant.

## Conclusions

Our results demonstrate the time- and dose-dependent anti-growth effects of IT anti-c-Met/PE38KDEL against GC cell lines. The anti-cancer effect of IT occurred primarily through inhibition of protein synthesis, and caspase-3-mediated apoptosis, suggesting the potential value of IT as an anti-c-MET therapeutics for GC.

## Abbreviations

IT: Immunotoxins; GC: Gastric carcinoma; HGF: hepatocyte growth factor; CCK8: Cell Counting Kit 8; FCS: fetal calf serum; TBS: Tris-buffered saline; IR: inhibition rate; PBS: phosphate-buffered saline; SDS: sodium dodecyl sulphate; PAGE: polyacrylamide gel electrophoresis.

## Competing interests

The authors declare that they have no competing interests.

## Authors' contributions

LZ AND XW: Conceived, designed, and coordinated the study and acquired the necessary funding; and carried out the majority of the in vitro studies. drafted the manuscript. CN and ZXJ: carried out all subsequent analyses; FXM: carried out some of the in vitro experiments; ZXH and FZQ: Contributed to the design and coordination of the study and aided with manuscript preparation. All authors read and approved the final manuscript.
